# Supplementary material for: Optimization of differentiation and transcriptomic profile of THP-1 cells into macrophage by PMA
Source: PLoS One. 2023 Jul 17;18(7):e0286056. doi: 10.1371/journal.pone.0286056 (PMC10351730; doi:10.1371/journal.pone.0286056)
Supplement: S1 File — (ZIP) [file pone.0286056.s001.zip › supporting files/RNA-seq report.zip!/RNA-seq report/4_GOEnrichment_result/induce_vs_control/Enrich_bar/induce_vs_control.DEG_down_GO_Enriched_bar.pdf]

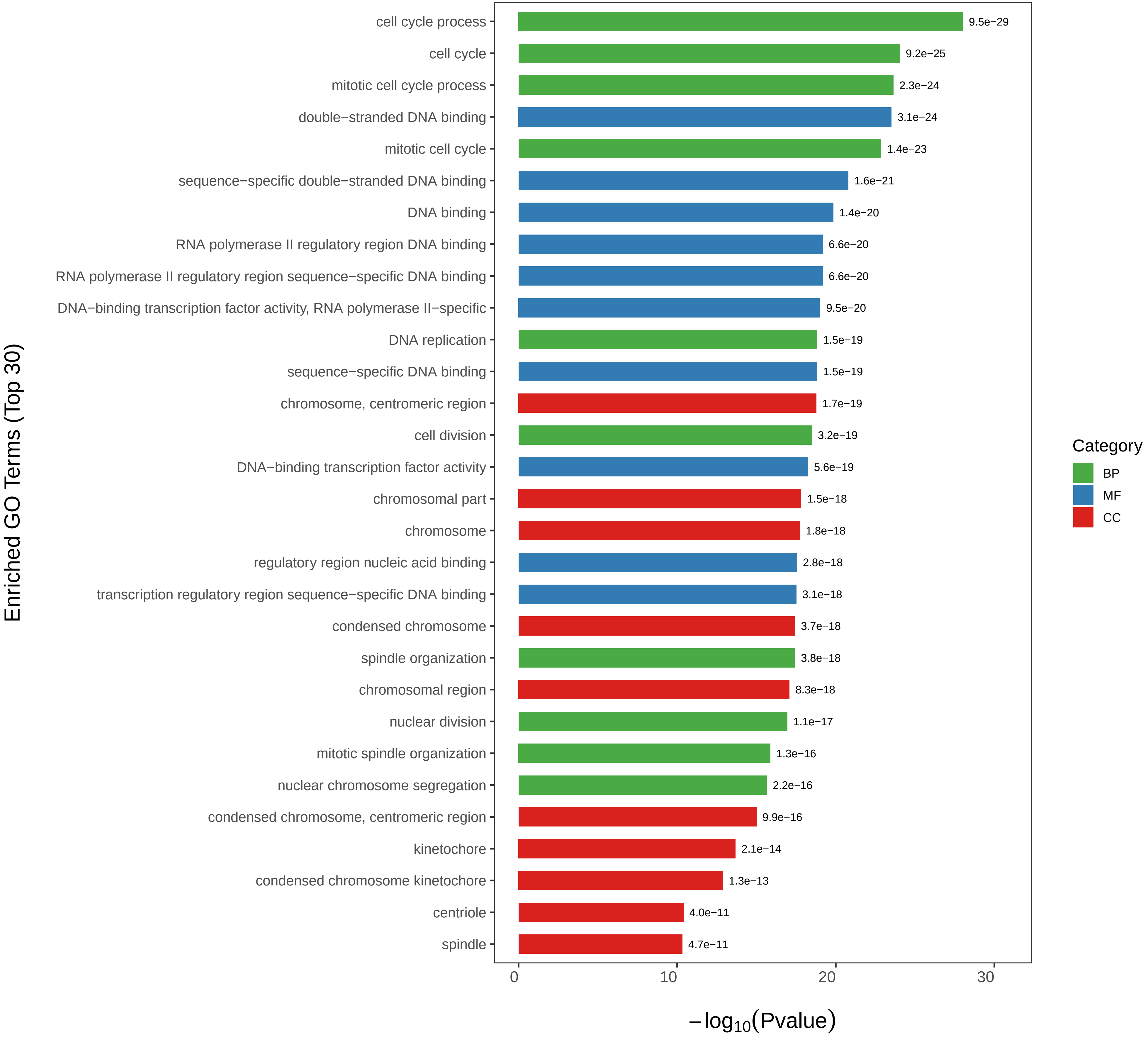

Enriched GO Terms (Top 30)
cell cycle process 9.5e−29
cell cycle 9.2e−25
mitotic cell cycle process 2.3e−24
double−stranded DNA binding 3.1e−24
mitotic cell cycle 1.4e−23
sequence−specific double−stranded DNA binding 1.6e−21
DNA binding 1.4e−20
RNA polymerase II regulatory region DNA binding 6.6e−20
RNA polymerase II regulatory region sequence−specific DNA binding 6.6e−20
DNA−binding transcription factor activity, RNA polymerase II−specific 9.5e−20
DNA replication 1.5e−19
sequence−specific DNA binding 1.5e−19
chromosome, centromeric region 1.7e−19
cell division 3.2e−19
DNA−binding transcription factor activity 5.6e−19
chromosomal part 1.5e−18
chromosome 1.8e−18
regulatory region nucleic acid binding 2.8e−18
transcription regulatory region sequence−specific DNA binding 3.1e−18
condensed chromosome 3.7e−18
spindle organization 3.8e−18
chromosomal region 8.3e−18
nuclear division 1.1e−17
mitotic spindle organization 1.3e−16
nuclear chromosome segregation 2.2e−16
condensed chromosome, centromeric region 9.9e−16
kinetochore 2.1e−14
condensed chromosome kinetochore 1.3e−13
centriole 4.0e−11
spindle 4.7e−11
0
10
20
30
$-\log_{10}(\text{Pvalue})$
Category
BP
MF
CC
